# Impact of road network structure on dementia-related missing incidents: a spatial buffer approach

**DOI:** 10.1038/s41598-020-74915-y

**Published:** 2020-10-29

**Authors:** Vaisakh Puthusseryppady, Ed Manley, Ellen Lowry, Martyn Patel, Michael Hornberger

**Affiliations:** 1 Norwich Medical School, 2.04 Bob Champion Research and Education Building, University of East Anglia, Norwich, NR4 7TJ UK; 2 Centre for Advanced Spatial Analysis, University College London, Gower Street, London, WC1E 6BT UK; 3 School of Geography, University of Leeds, Leeds, LS2 9JT UK; 4 School of Psychology, University of East Anglia, Norwich, NR4 7TJ UK; 5 Norfolk and Norwich University Hospitals National Health Service (NHS) Foundation Trust, Colney Lane, Norwich, NR4 7UY UK

**Keywords:** Alzheimer's disease, Signs and symptoms

## Abstract

Dementia-related missing incidents are a highly prevalent issue worldwide. Despite being associated with potentially life-threatening consequences, very little is still known about what environmental risk factors may potentially contribute to these missing incidents. The aim of this study was to conduct a retrospective, observational analysis using a large sample of police case records of missing individuals with dementia (n = 210). Due to the influence that road network structure has on our real world navigation, we aimed to explore the relationship between road intersection density, intersection complexity, and orientation entropy to the dementia-related missing incidents. For each missing incident location, the above three variables were computed at a 1 km radius buffer zone around these locations; these values were then compared to that of a set of random locations. The results showed that higher road intersection density, intersection complexity, and orientation entropy were all significantly associated with dementia-related missing incidents. Our results suggest that these properties of road network structure emerge as significant environmental risk factors for dementia-related missing incidents, informing future prospective studies as well as safeguarding guidelines.

## Introduction

Dementia-related missing incidents are a highly prevalent yet poorly understood phenomenon worldwide. As per definition, dementia-related missing incidents occur when a person with dementia is not at an expected location and their whereabouts are unknown to their carer^1^. It has been reported that 70% of people with dementia may experience at least one missing incident at any stage of the disease, with some even at risk for going missing multiple times^2–5^. At present there are an estimated 40,000 people with dementia that go missing for the first time every year in the UK—a figure that is likely to grow with the projected increase in the dementia population worldwide^2,6^.

Although the timing of missing incidents can be highly unpredictable, it has been reported that they most often arise when there is a temporary gap in supervision of the person with dementia from their carer such as when this individual leaves the house to perform a daily activity (i.e., neighbourhood walks, going to the shop, etc.) or even in the middle of the night when the carer is sleeping^7,8^. Being prevalent worldwide, dementia-related missing incidents are indeed associated with negative consequences for the person with dementia including causing them to suffer from a reduced sense of autonomy, increasing their likelihood of being admitted to a care home by seven times, as well as increasing their risk of sustaining harm^4,9^. It also has a negative impact on the wider community as it not only increases carer burden but also often elicits the involvement of law enforcement groups (i.e., the police) as well as community search resources^8,10–12^. Despite the widespread nature of dementia-related missing incidents and its resulting consequences, very little is still known about the aetiology of people with dementia going missing. Previous work has shown that dementia-related missing incidents are most commonly seen in Alzheimer’s Disease (AD) when compared to other dementias^13,14^. At a brain level, it has been suggested that people with AD exhibit neuropathology induced alterations to the navigation network in the brain. Specifically, alterations to the medial temporal and parietal lobe structures lead to respective impairments in egocentric (body-based) and allocentric (map-based) navigation, as well as the interplay between the two strategies^15^. Indeed, such impairments in spatial navigation abilities can often lead to people with dementia making wayfinding errors when navigating outdoors, which they are ultimately unable to recover from and as a result go missing.

In particular, previous research has speculated that there may be some external factors which potentially act as triggers for people with dementia to make wayfinding errors that lead to them going missing^1^. Considering the key role that the environment plays in real world navigation^16^, whether specific features of the environment act as such triggering factors warrants investigation. Surprisingly however, there have been almost no studies reporting real world environmental risk factors for dementia-related missing incidents. Due to the unpredictable nature of these incidents^1,8^, identifying environmental risk factors is of importance as it can potentially help identify/predict areas where people with dementia may be at a high risk to go missing from. Clearly, this knowledge can further understanding of why people with dementia go missing as well as contribute to the development of safeguarding guidelines to prevent them from going missing in the future.

One of the key environmental variables that guide and influence human navigation behaviour in the real-world is the structure of road networks^17^. Previous studies have investigated the impact of road network structure on wayfinding behaviour^18,19^, but not in the context of dementia-related missing incidents. In the current study, we explore the relationship between road network structure and dementia-related missing incidents using a spatial buffer approach. Specifically, we investigate the roles that road intersection density, intersection complexity, and orientation entropy may play in causing people with dementia to go missing. The first two variables are of interest as road intersections represent locations in the environment where crucial navigation decisions must be made^20,21^, and as such are clearly relevant for navigation. Meanwhile, the third variable is of interest as it measures the orientation of roads within a given area of the environment, and in doing so informs us of how ordered (or disordered) the overall layout of the road network is within these areas^22^. To this end, we conducted a retrospective analysis of records of dementia-related missing incidents in a single region of the UK over a 3-year period. We hypothesize that higher road intersection density would lead to increased missing incidents, as the more frequently the people with dementia have to make critical navigation decisions, the more likely they are to make an error and make a wrong turn. We also predict that higher road intersection complexity would lead to increased missing incidents, as the more route options an intersection has, the harder it will be for the people with dementia to identify and select the correct route. Lastly, we hypothesize that higher road orientation entropy would also be associated with increased missing incidents, as road networks with a high entropy would be less ordered in structure and hence more complex to navigate through.

## Methods

### Study design

This study was conducted using records of missing people with dementia provided by the Norfolk police. The records contained a total of 210 anonymised cases for the Norfolk County (total population 898,390) in the United Kingdom (UK), covering dates from January 2014 to December 2017.

Each missing person with dementia case contained the following variables—“date missing, gender, age, location missing from (town and postcode), type of setting missing from (care home/hospital, domestic residence, public), location found (building name/road and town), case details (circumstances in which the individual went missing/was found), time missing (minutes), and whether it was the first time missing (yes/no)”. For each case, the location they went missing from was classified as urban or rural using the UK Office for National Statistic’s 2011 rural urban classification guide^23^. Moreover, from the case details it was inferred as to whether each individual had sustained harm (i.e., injuries/death) during the missing incident.

Using the above variables, we investigated retrospectively if there were any demographic risk factors for the missing people with dementia as well as the impact of outdoor landmark density in causing these people to go missing in a previous study^24^. Here, using the same dataset, we are investigating the impact of road network structure in causing people with dementia to go missing.

Ethical approval for this study was granted by the Faculty of Medicine and Health Sciences Research Ethics Committee at the University of East Anglia (Ref. FMH2017/18–94), and all research was conducted in accordance with the relevant guidelines and regulations.

### Demographics analysis

The missing people with dementia data contained both continuous and categorical variables. We analysed the demographics of this data in a previous study, where we explored gender differences in the missing incident variables and risk factors for going missing multiple times as well as sustaining harm whilst missing (for details—see^24^).

### Missing incidents and intersection density

**Figure 1:**
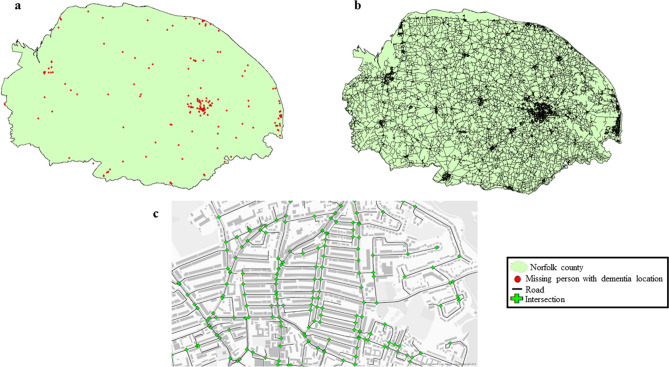
(**a**) Missing person with dementia locations in Norfolk. (**b**) Road network dataset overlaid onto map of Norfolk. (**c**) Roads and intersections in the road network dataset (the maps/satellite imagery in the figure were created in ArcGIS software version 10.6.1, using publicly available shape-files from Ordnance Survey (https://www.ordnancesurvey.co.uk/business-government/products/open-map-roads) and the UK Data Service (https://census.ukdataservice.ac.uk/get-data/boundary-data.aspx). Contains OS data © Crown copyright and database right 2020; Contains National Statistics data © Crown copyright and database right 2020).

We first explored the impact of road intersection density in causing people with dementia to go missing. For this we first plotted out the locations these individuals went missing from onto a map of Norfolk, in shape-file format, on ArcGIS software version 10.6.1^25^ (Fig. 1a). As the locations were reported as postcodes in the dataset, for the purpose of this analysis the centroid of the reported postcodes were taken for these locations. In total, the 210 cases mapped onto 168 different locations across the region, with there being 17 locations were multiple individuals went missing. For individuals reported as having gone missing multiple times, only the location of their most recent incident was reported. Moreover, there were 3 cases where the location the individual went missing from was not reported. The road network data used in this study was the Ordnance Survey Open Roads layer (https://www.ordnancesurvey.co.uk/business-government/products/open-map-roads), containing all the roads (major and minor) and intersections in the UK. In this dataset, road intersections were represented as vertices and the roads themselves were represented as edges connecting the vertices (Fig. 1c). Here, all roads and intersections for the Norfolk region were extracted and overlaid onto the map of Norfolk (Fig. 1b).

The measure of intersection density was employed at a spatial buffer level. This approach involves generating a buffer zone of a specific radius around each missing person with dementia location and identifying the number of intersections that fall within these zones. Since we do not have any trajectory data for the missing people with dementia, employing a 1 km radius buffer zone enables us to take into account any direction that these individuals could have travelled and as such, allows us to estimate all potential intersections that they could have encountered at the time and place they went missing. A radius of 1 km was chosen for the buffer zones as according to previous health geography studies^26–28^, this has been suggested to be an appropriate distance to capture all environments accessible within a reasonable walking distance from a particular location. Moreover, since a radius of 1 km was used in our previous study which also utilised a spatial buffer approach, we decided to keep using this distance here to ensure consistency with our previous work^24^.

**Figure 2:**
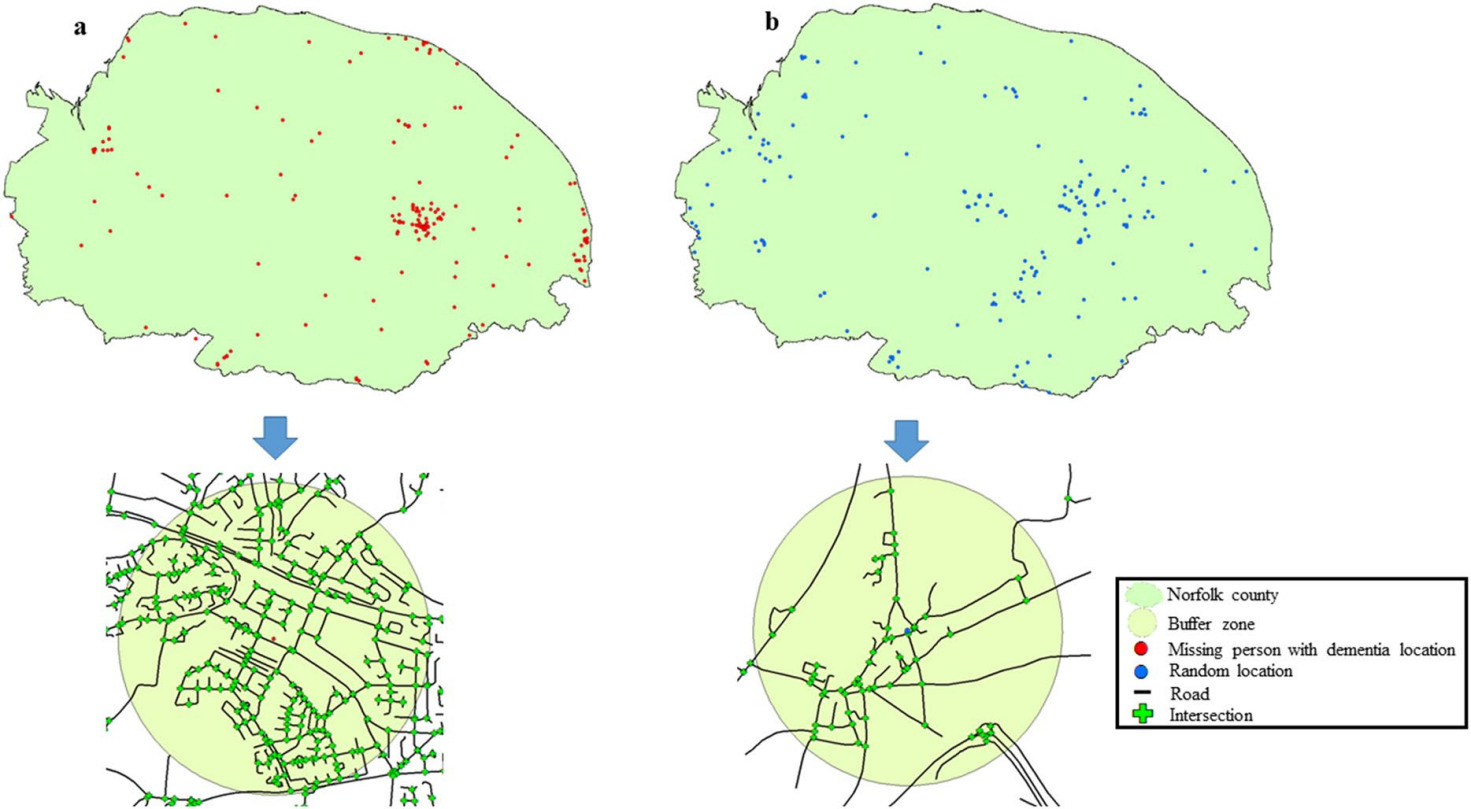
(**a**) Road intersections falling within a 1 km radius buffer zone of a single missing person with dementia location (urban region, residential land). (**b**) Road intersections falling within a 1 km radius buffer zone of a single random location (urban region, residential land) (the maps/satellite imagery in the figure were created using ArcGIS software version 10.6.1, using publicly available shape-files from Ordnance Survey (https://www.ordnancesurvey.co.uk/business-government/products/open-map-roads) and the UK Data Service (https://census.ukdataservice.ac.uk/get-data/boundary-data.aspx). Contains OS data © Crown copyright and database right 2020; Contains National Statistics data © Crown copyright and database right 2020).

Here, geodesic buffer zones with a radius of 1 km was generated for each of the 168 missing person with dementia locations (Fig. 2a), and the intersection density within each buffer zone was computed. Following this, we generated a set of 168 random, control locations across the entire Norfolk region using an in-built algorithm in ArcGIS (Fig. 2b). These random locations had a similar urban/rural distribution as the missing person with dementia locations as well as fell in the same types of land (for details, see^24^). Similar to the missing person with dementia locations, we generated geodesic buffer zones with a radius of 1 km for each of the 168 random locations, and computed the intersection density within these buffer zones.

As the intersection density within the buffer zones of both the missing person with dementia and random locations groups had a non-normal distribution, a Wilcoxon Rank Sum Test was run to compare this variable in both groups.

### Missing incidents and intersection complexity

We next explored the complexity of the road intersections at the missing person with dementia and random locations. Here, intersection complexity refers to the number of route options that branch out from a single intersection. For example, intersections with 5 route options would be considered to be more complex than intersections with only 2 route options. For this, we computed the average intersection complexity exhibited in each of the missing person with dementia and random location buffer zones. Wilcoxon Rank Sum Tests were then run to compare this variable in both groups.

### Missing incidents and road orientation entropy

We lastly explored the impact of road orientation entropy in causing people with dementia to go missing. Here, road orientation entropy refers to a measure of how ordered or disordered the overall layout of a road network within a given area is.

**Figure 3:**
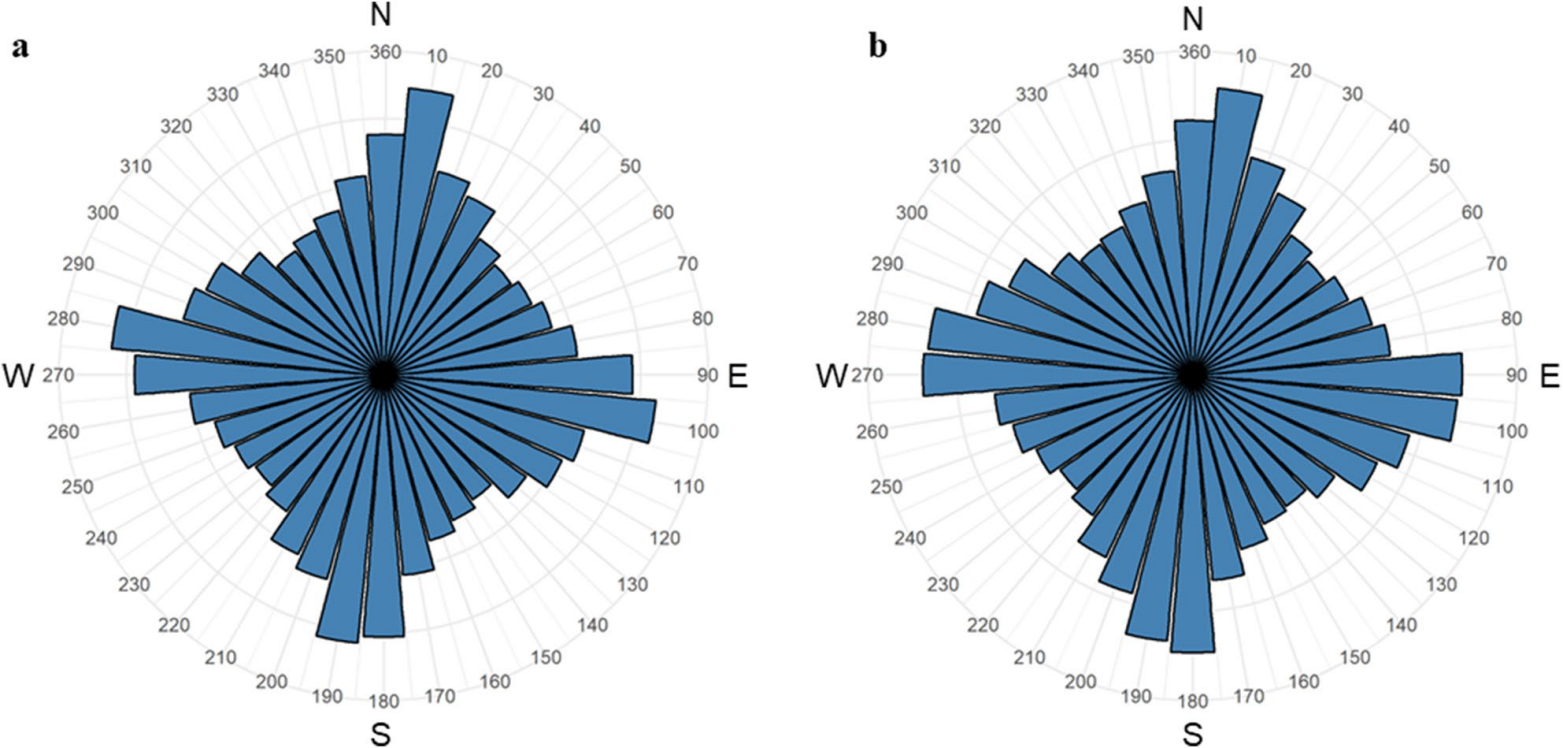
Rose diagrams showing the orientations of roads in a single (**a**) Missing person with dementia location buffer zone (urban, residential area) and (**b**) random location buffer zone (urban, residential area). The direction of the bars represent the orientation of the roads, whilst the height of the bars represent the frequencies of roads exhibiting that orientation.

We first calculated the angular orientation of each road in the missing person with dementia and random location buffer zones. Since each road is bidirectional in nature, this was done by measuring the angle between compass North and the start/end points of the road respectively. Hence for each road this yielded two angles that were reciprocals of one another (i.e., If start point of road had orientation angle of 60°, the end point would have angle of 300°). After calculating the orientation of all roads in the missing person with dementia and random location buffer zones, we group these values into 36 bins, with each bin representing incremental ranges of 10° (i.e., 0–10, 11–20, 21–30…351–360) (Fig. 3).

We next calculated Shannon’s entropy (H)^29^ for the distribution of road orientations across all bins for the missing person with dementia and random location buffer zones, using the formula:

$$\mathrm{H}= -\sum_{i=1}^{n}\mathrm{P}\left({0}_{i}\right) {\mathrm{log}}_{e} \mathrm{P}({0}_{i})$$

where *n* is the total number of bins, *i* is the bin number, and P(0_*i*_) is the probability of a randomly selected road from the sample falling in bin number *i*. In essence, the entropy measure tells you how ordered the layout of the roads in each buffer zone are, with higher entropy indicating low order and lower entropy indicating high order.

### Missing incidents and intersection density, intersection complexity, and orientation entropy—multiple regression modelling

To explore whether road intersection density, intersection complexity, or orientation entropy was a better predictor for missing people with dementia across Norfolk, we ran ordinarily least square multiple regressions.

**Figure 4:**
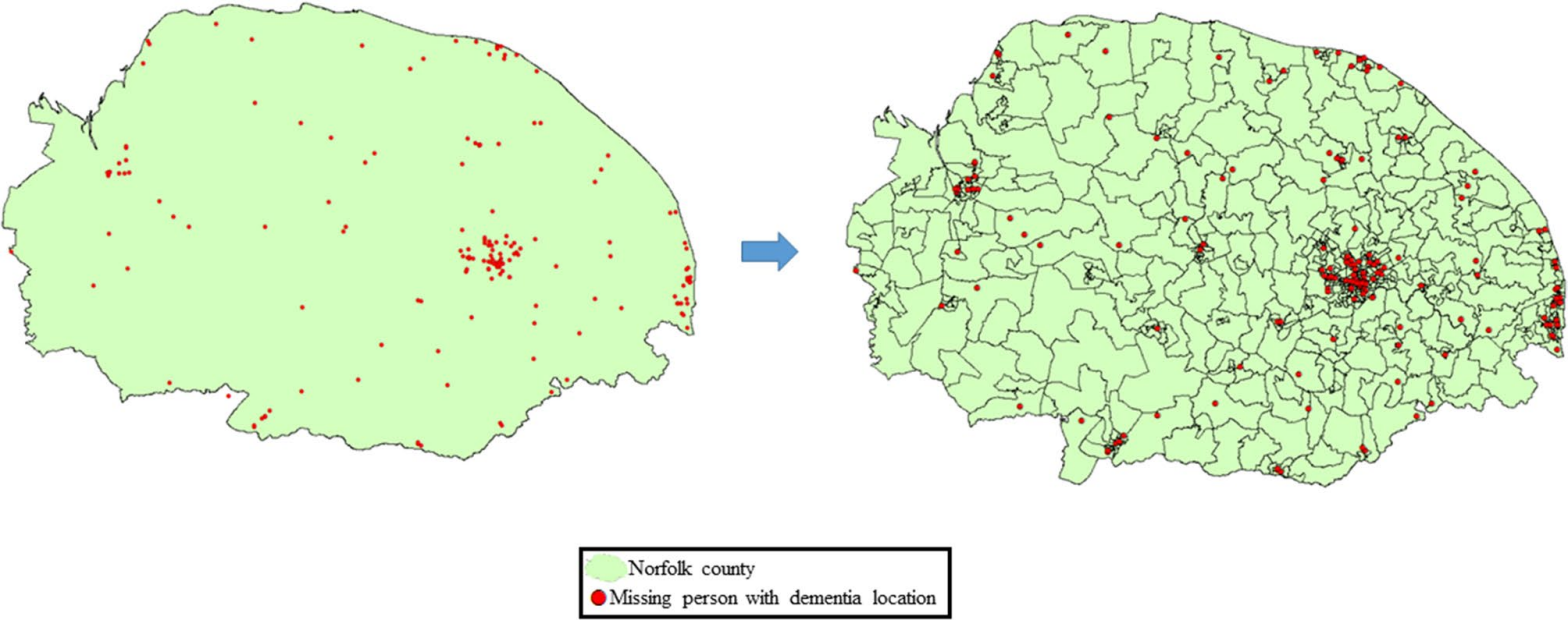
Map of Norfolk containing all the missing person with dementia locations, sub-divided into its different LSOAs (the maps/satellite imagery in the figure were created using ArcGIS software version 10.6.1, using publicly available shape-files from the UK Data Service (https://census.ukdataservice.ac.uk/get-data/boundary-data.aspx). Contains OS data © Crown copyright and database right 2020; Contains National Statistics data © Crown copyright and database right 2020. Part of this figure has been reprinted from Puthusseryppady et al.^24^, Copyright (2019), with permission from IOS Press. The publication is available at IOS Press through https://doi.org/10.3233/JAD-190244).

To provide specific spatial units for the analysis, the Norfolk county was sub-divided into its lower layer super output areas (LSOA) (Fig. 4). These are geographic units that are commonly used by the UK Office for National Statistics for reporting small area statistics (eg. neighbourhood population, income estimates, housing etc.)^30^. LSOAs were chosen as our spatial units of analysis due to their good ecological validity in allowing the data to be split into three main localities (urban, rural town and rural villages). For this, we downloaded a shape-file containing the UK sub-divided into its different LSOAs from the UK Office for National Statistics Open Geography Portal^31^, and extracted only the LSOAs covering the Norfolk region. In this shape-file, each LSOA was classified as being either urban or rural based on population density and the latter were further sub-classified into rural towns and rural villages based on household density^23^. All the 168 missing person with dementia locations were then aggregated into the respective LSOAs in which they fell in, with 96 locations falling within urban LSOAs, 33 in rural town LSOAs, and 39 in rural village LSOAs. To control for the distribution of population densities across Norfolk, the number of missing people with dementia falling within each LSOA was normalised for the total population of that LSOA. Moreover, LSOAs that did not exhibit a missing person with dementia were removed from the analysis (Fig. 4).

Ordinary least squares multiple regression models were then run on the remaining LSOAs where the number of missing people with dementia in each LSOA were regressed against the intersection density, average intersection complexity, and road orientation entropy of each LSOA. In total, three multiple regression models were run—one for urban, rural town, and rural village regions respectively.

All regression models were run in R software package version 3.4.2^32^.

## Results

### Demographics risk factors

All results of the demographics analysis were conducted and reported in a previous study (for details—see^24^). However, of relevance to this study it is important to note that a similar number of males and females went missing. Most people went missing from domestic residence settings (*n* = 134), followed by care facilities (*n* = 52) and general public locations (*n* = 23). Subgroups of missing people with dementia that went missing multiple times (*n* = 52), as well as those that sustained harm during the missing incident (*n* = 10) were also identified (Table 1). All missing people with dementia were found alive except for one case.

**Table 1:** Demographics of the missing people with dementia.

	Total
Cases	210
Males/females	114/96
Age (median)	81
**Setting missing from**	
Domestic residence	134
Care facility	52
Public place	23
**Locality missing from**	
Urban	116
Rural town	37
Rural village	54
Unspecified	3
Distance travelled (median; meters)	2000
Time missing (median; minutes)	55.5
Missing multiple times	52
Sustained harm	10

### Missing incidents and intersection density, complexity

There was a significantly higher intersection density within the missing person with dementia location buffer zones when compared to the random location buffer zones (W = 21,425, p < 0.001). In addition, the average intersection complexity in the missing person with dementia location buffer zones were also significantly higher when compared to that of the random location buffer zones (W = 16,522, p = 0.006).

### Missing incidents and road orientation entropy

There were no significant differences in the orientation entropy of roads in the missing person with dementia location buffer zones when compared to that of the random location buffer zones (W = 15,482, p = 0.081). However, considering that this p value of 0.081 indicates a statistical trend towards significance, it may very well be that 1 km may have the limitation of being too small a radius (for the buffer zone) to fully capture differences in the orientation of roads between locations. Hence as an exploratory measure, we expanded the buffer zone radius to 2 kms for all missing person with dementia and random locations, and ran the analysis again. Here, we found that the roads in the missing person with dementia location buffer zones had a significantly higher orientation entropy than the roads in the random location buffer zones (W = 16,352, p = 0.012).

### Missing incidents and intersection density, intersection complexity, and road orientation entropy—multiple regression modelling

The multiple regression modelling showed that in urban regions, increased intersection density was a significant predictor for increased missing people with dementia (p < 0.05) whilst neither intersection complexity nor road orientation entropy were significant predictors (p = 0.1846; p = 0.9496) (r^2^ = 0.05545). Meanwhile, neither intersection density, intersection complexity, nor road orientation entropy were significant predictors for missing people with dementia in either rural towns or villages.

## Discussion

In line with the hypothesis, our results showed that increased intersection density and complexity were associated with the missing incidents. However, our hypothesis that increased road orientation entropy would also be associated with the missing incidents was true only when using a 2 km radius buffer zone, and not 1 km.

Our results overall suggest that increased intersection density, intersection complexity, and road orientation entropy may all be environmental risk factors causing people with dementia to go missing. To the best of our knowledge, this is the first study to report the influence of road network structure in causing people with dementia to go missing in the community. Previously, only one study has been conducted looking at the relationship between roads and dementia, however this study was focused on the incidence of dementia as opposed to the prevalence of people with dementia that go missing^33^.

Due to the retrospective nature of the data, the exact mechanisms underlying how increased intersection density, intersection complexity, and road orientation entropy contribute to cause people with dementia to go missing is at present unclear. Considering that intersections represent spatial decision points along a route, navigating through environments that are rich in intersections would more often place people with dementia in situations where important navigation decisions must be made (‘which way do I turn here?’). This in conjunction with the presence of various route options at the intersections has the potential to challenge the already impaired spatial navigation abilities of these individuals^15^, increasing their chances of making an error along a journey and ultimately getting lost. This may especially be true when errors accumulate over multiple, sequential intersections—making it more difficult for the people with dementia to navigate to their intended location.

Our second set of results showed a significant association between increased road orientation entropy and people with dementia that went missing. The roads surrounding the missing person with dementia locations exhibited a higher orientation entropy than those surrounding the random locations, thereby indicating that the roads surrounding the former have relatively less-defined patterns when compared to the latter. It has previously been shown that people remember roads with well-defined patterns (i.e., more grid-like) better than roads that have less-defined patterns (i.e., less grid-like) in their cognitive maps of local environments^34^. Considering this and the fact that people with AD are widely reported to have impairments in map-based (allocentric) navigation^15^, it can be speculated that these individuals may lose earlier the parts of their cognitive maps containing roads with less-defined patterns, causing them to feel disoriented in these environments and contributing to them eventually going missing.

Despite our results showing a significant association for intersection density, intersection complexity, and road orientation entropy at a buffer level, at a LSOA level we found that increased intersection density was the sole significant predictor for increased missing incidents, and that too only in urban regions and not in rural towns or villages. Indeed, there may be other, more significant variables that may be predictive of missing incidents in rural regions, which requires further investigation.

Overall, our findings suggest that pockets of regions with a high road intersection density, intersection complexity, and orientation entropy could represent likely locations where a missing incident could occur for people with dementia . This is indeed a factor that should be considered by carers and healthcare professionals, whereby it may especially be beneficial to plan and use routes with fewer intersections (where possible) on independent journeys or recommend GPS tracking devices in areas exhibiting complex road network configurations. Moreover, with increasing efforts being made to build dementia friendly communities, especially in town planning, the structure of the road networks should be a factor that should also be taken into account when planning roadways in these communities. Many residential areas have irregular road layout patterns that may not necessarily be designed accounting for the navigation difficulties seen in people with dementia^35^. Indeed, it may be useful to design roads of neighbourhoods in areas with a high older population density to be more straight/ordered (i.e., grid-like) but with fewer and more simple intersections. Doing so would make these environments easier to navigate for people with dementia by offering more direct and continuous routes to local amenities^35,36^. This road design could have potential advantages by not only helping to reduce the risk of people with dementia getting lost, but also helping carers to find them in the event that they do go missing. Ultimately, this could lead to people with dementia getting outdoors more often and hence increasing their overall quality of life.

Despite these exciting and novel findings, our study has some noteworthy limitations. First and foremost, the missing person with dementia locations reported in the data are the last known location of the person with dementia by the carer and may not in fact represent the true location in which the individual went missing from. Moreover, due to the retrospective nature of the data it is not possible for us to know the exact roads that the people with dementia took on the journey in which they went missing as well as the exact intersections they may have come across during this journey. Future studies should investigate prospectively the effect that road network structure has in causing people with dementia to go missing using trajectory data. Lastly, the sample size of the study only represent missing incidents that were reported to the police, which mostly occurs in the more severe cases (i.e., when the family or neighbours cannot locate the missing person with dementia themselves). It is therefore highly likely that the true prevalence rates of missing people with dementia are much higher and occur in far more locations across the county than reported. As such, the findings of this study need to be replicated with more representative samples of missing incidents in the future.

In conclusion, we provide novel evidence for road intersection density, intersection complexity, and orientation entropy being important environmental risk factors for dementia-related missing incidents. The results of this study provide a platform for future studies to investigate this variable more systematically and can potentially help contribute to the development of safeguarding guidelines to help prevent people with dementia from going missing in the community.

## Data Availability

The road network dataset analysed in the current study is publicly available from Ordnance Survey (https://www.ordnancesurvey.co.uk/business-government/products/open-map-roads). The LSOA shape-file for the UK dataset used in the current study is publicly available from the UK Office for National Statistics Open Geography Portal (https://geoportal.statistics.gov.uk/datasets/da831f80764346889837c72508f046fa_2?geometry=-22.104%2C50.43%2C19.819%2C55.078). The missing people with dementia police records dataset analysed in the current study are available from the Norfolk Constabulary Team but restrictions apply to the availability of these data, which were used under special permission for the current study, and so are not publicly available. The data is however available from the authors upon reasonable request and with permission of the Norfolk Constabulary Team.
